# Quercetin inhibits the progression of endometrial HEC-1-A cells by regulating ferroptosis—a preliminary study

**DOI:** 10.1186/s40001-022-00934-2

**Published:** 2022-12-15

**Authors:** Xiaoqin Li, Qianqian Zhu, Meng Ma, Haiyan Guo

**Affiliations:** 1 Biobank of Ninth People’s Hospital, Shanghai Jiao Tong University School of Medicine, No. 115, Jinzun Road, Shanghai, 200011 China; 2 Department of Assisted Reproduction, Shanghai Ninth People’s Hospital, Shanghai Jiao Tong University School of Medicine, Research Center for Specialty Strategy of China Hospital Development Institute, Shanghai Jiao Tong University, Shanghai, 200011 China

**Keywords:** Endometrial carcinoma, Quercetin, Ferroptosis, Mechanism

## Abstract

**Background:**

Endometrial carcinoma (EC) is one of the most common female reproductive system tumors, which seriously threatens women's health. This preliminary study aimed to investigate the effects of quercetin on the EC cells and explore the potential mechanism.

**Methods:**

In this study, the effects of quercetin on endometrial cancer HEC-1-A cells were studied by a series of cell biological methods, including CCK-8 detection of cell activity, Western blotting of ferroptosis-related proteins, apoptosis detection, reactive oxygen species (ROS) detection and other detections.

**Results:**

Our results showed that quercetin inhibited the proliferation and migration of EC cells, induced cell apoptosis, and affected the cell cycle. Furthermore, the anti-tumor effect of quercetin was related to the induction of ferroptosis in the EC cells.

**Conclusions:**

Our study shows quercetin may exert anti-tumor effects, which may be related to the regulation of ferroptosis. Our study provides evidence for the future treatment of EC with small molecule drugs.

## Background

Endometrial carcinoma (EC) is one of the most common female reproductive system tumors, and there are nearly 200,000 new cases of EC each year [1]. EC accounts for approximately 76,000 deaths worldwide each year, and its incidence is still increasing over year [2–4]. EC is a group of epithelial malignancies occurring in the endometrium, especially in the perimenopausal and postmenopausal women. The main symptoms are abnormal uterine bleeding, abnormal vaginal drainage, pain and mass in the lower abdomen [5, 6]. Currently, the treatment of choice for EC is still surgery. Chemotherapy and/or radiotherapy serve as adjunctive therapies for patients at moderate to high risk of recurrence. Although these traditional treatments are effective, there are still serious side effects associated with them like hepatic and renal toxicities gastrointestinal toxicity, bone marrow suppression, irregular menstruation, and so on [7]. In addition, long-term treatment will also induce chemotherapy resistance, affecting the therapeutic efficacy [8, 9]. Therefore, it's important to develop new treatments for EC.

**Fig. 1:**
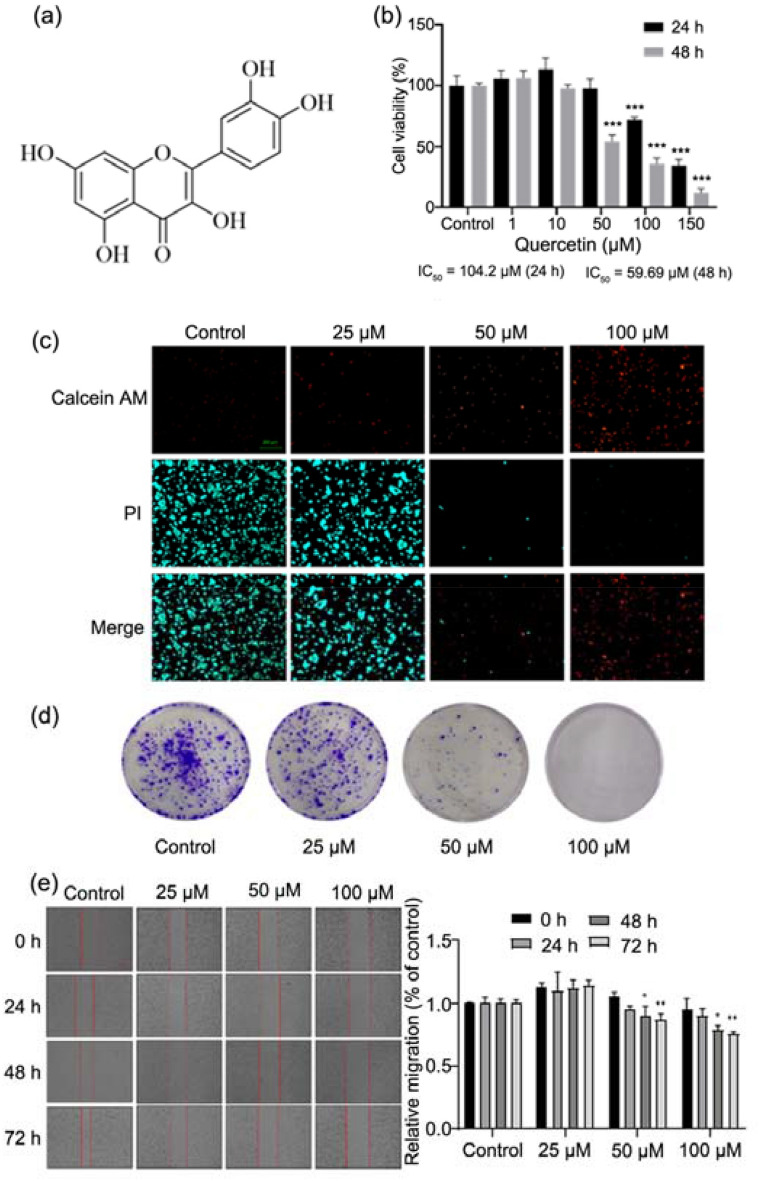
Quercetin inhibited proliferation and migration of HEC-1-A cells. **a** Structure of quercetin. **b** Effect of quercetin on the proliferation of HEC-1-A cells. Cells were treated with quercetin for 24 or 48 h, then CCK-8 assay was used to detect the cell proliferation rate (%), and the IC_50_ was calculated. **c** PI/Calcein AM staining for HEC-1-A cells. Cells were treated with quercetin for 24 h, then stained and visualized under a fluorescent microscope. **d** Cell colony formation assay for HEC-1-A cells after treatment with quercetin at different concentrations. **e** Cell scratch assay for HEC-1-A cells. Cells were treated with quercetin for 72 h and photographed at 0, 24, 48 and 72 h. Data are expressed as mean ± SD (*n* = 3). ***p < 0.001 vs. Control

As multi-pathway, multi-target and highly effective anticancer adjuvant drugs with less toxic and side effects, Traditional Chinese Medicine and natural products have been widely used in studies on the treatment of cancers in recent years. Studies have confirmed that the Western medicines combined with Traditional Chinese medicine may exert synergistic effects to improve the quality of life of patients [10]. Quercetin is a natural flavonoid separated from the flowers, leaves and fruits of many plants. It is also known as 3, 3’, 4, 5, 7-pentahydroxy flavonoid with the molecular weight of 302.24 (Fig. 1a). To date, it has been found that quercetin possesses a variety of pharmacological activities, such as antioxidative, free radical scavenging, anti-inflammatory, antibacterial, antiviral, anti-tumor, gastric protection, and immune regulatory activities, and has been used in the treatment of obesity and cardiovascular diseases [11–16]. However, few studies have been conducted to investigate the biological effects of quercetin on the EC cells. This study was undertaken to investigate the effects of quercetin on the proliferation, migration, apoptosis and cell cycle of EC HEC-1-A cells and explore the potential molecular mechanism. Our study will provide evidence for the development of new drugs for the clinical treatment of EC.

In the past decades, several types of regulated cell death (RCD) have been identified and defined according to their different morphological characteristics, biomarkers, or regulatory mechanisms [17–19]. They include apoptosis, necroptosis, pyroptosis and autophagy. Ferroptosis is also a form of RCD driven by the iron-dependent accumulation of lipid hydroperoxides [20]. Increasing evidence suggests that ferroptosis is involved in both normal physiology and pathology in mammals, including degenerative diseases, ischemia–reperfusion injury, and carcinogenesis [21]. Some studies have suggested that ferroptosis may be a natural tumor suppressive mechanism, and thus targeting ferroptosis becomes a promising therapeutic strategy for the treatment of cancers [22, 23]. A variety of studies have confirmed that quercetin may regulate the iron metabolism to exert important biological effects [24, 25]. Quercetin can reduce high-valent iron, inhibit lipid oxidation and suppress iron-catalyzed reactive oxygen species (ROS) production [26]. As an excellent antioxidant, its antioxidative activity is mainly manifested by affecting glutathione, enzyme activity and ROS production and by regulating signal transduction pathways such as nuclear factor-kappa B (NR-κB), mitogen-activated protein kinase (MAPK) and AMP-activated protein kinase (AMPK) pathways [27].

In the present study, the effects of quercetin on the malignant behaviors of EC cells were investigated, and the potential role of ferroptosis in the biological effects of quercetin on the EC cells was further explored. Our findings may provide evidence for the treatment of EC.

## Methods

### Chemicals and reagents

Quercetin (Q4951, purity > 95%, Sigma-Aldrich), erastin (HY-15763, purity > 99% MedChemExpress) and ferrostatin-1 (SML0583, purity > 95%, Sigma-Aldrich) were dissolved in DMSO. For the cell experiments, quercetin was dissolved in DMSO to achieve the pre-designed concentration and cells were treated with quercetin for 24 h or 48 h. The following kits were used in the present study: Cell-Counting-Kit-8 (CCK-8) kit, DAPI Staining Solution, Crystal Violet Staining Solution, Calcein/PI Live/Dead Viability/Cytotoxicity Assay kit, Cell Cycle and Apoptosis Analysis kit, Cell Cycle and Apoptosis Analysis kit, mitochondrial membrane potential assay kit with JC-1 (Beyotime, China); Reactive Oxygen Species Assay kit (YEASEN, China). The antibodies used in this study included primary antibodies to anti-p53, anti-xCT, anti-glutathione peroxidase 4, anti-aconitase 1 (ACO1), anti-transferrin receptor, anti-ferritin light chain and anti-collagen IV (Abcam, USA); GAPDH (D16H11) XP rabbit mAb, anti-rabbit IgG, horse radish peroxidase (HRP)-linked antibody, anti-mouse IgG, and HRP-linked antibody (Cell Signaling Technology, USA).

### Cell culture

HEC-1-A cells were purchased from the National Collection of Authenticated Cell Cultures (Shanghai) and maintained at 37 °C under 5% CO_2_ in McCoy’s 5A medium (GNM16600, GENOM, China) supplemented with 10% fetal bovine serum and 1% penicillin–streptomycin solution.

### CCK-8 assay

Cells were seeded into 96-well plates at a density of 8 × 10^3^ cells/well and grown in McCoy’s 5A medium (plus 10% FBS). Cells in each well were treated with quercetin at different concentrations (0, 10, 20, 40, 80, and 160 μM, *n* = 5) for 24 or 48 h after the cell confluence reached about 100%. Then, these cells were incubated with CCK-8 solution to determine the cell proliferation rate (%). The optical density (OD) was measured at 450 nm in a microplate reader (Cytation 1, BioTek) for further IC50 analysis.

### Cell colony formation assay

Cells were seeded in 6‑well plates at a density of 1 × 10^3^ cells/well (2 ml/well) and then treated with quercetin at different concentrations (0, 25, 50 and 100 μM). The medium was refreshed once every 3 days. Cells were routinely maintained for about 2 weeks. When the colonies were visible, the medium was removed, and cells were rinsed with PBS and then fixed in 4% formaldehyde for 15 min. The supernatant was removed, and cells were stained with 0.25% crystal violet for 20 min and slowly rinsed with PBS. Finally, representative photos were captured and the cell colonies were counted.

### Cell scratch assay

Cells were seeded in 6‑well plates at a density of 6 × 10^5^/well (2 ml/well). A straight line was made in each well with a 1-ml pipette tip, and the cell debris were removed by washing with PBS. Then, cells were treated with quercetin at different concentrations (0, 25, 50 and 100 μM) at 37 °C in 5% CO_2_. The wound was captured at 0, 24, and 48 h under an inverted microscope (ZEISS, Axiocam 506 color).

### Western blotting

HEC-1-A cells were treated with quercetin at different concentrations for 24 h. The cells were lysed with NP-40 lysis buffer (Beyotime) containing 1 mM phenylmethanesulfonyl fluoride (PMSF) protease inhibitor (Beyotime) and 1 mM phosphatase inhibitor (Roche) on ice for 30 min, followed by centrifugation at 12,000 rpm for 20 min at 4 °C. The supernatant was collected and the protein concentration was measured using BCA Protein Assay Kit (Beyotime). Then, 20 μg of proteins was subjected to 10% SDS-PAGE and subsequently transferred onto PVDF membrane. The membrane was incubated with 5% non-fat milk in TBST at room temperature for 1 h. After rinsing thrice with TBST solution, the membrane was incubated with primary antibodies: anti-p53 (ab26; 1:1000), anti-xCT (ab37185; 1:1000), anti-aconitase 1 (ACO1) (ab236773; 1:1000), anti-transferrin receptor (ab84036; 1:1000), anti-ferritin light chain (ab69090; 1:1000), anti-collagen IV (ab6586; 1:1000), anti-glutathione peroxidase 4 (ab125066; 1:1000), or GAPDH (D16H11) XP rabbit mAb (1:2000, 5174, Cell Signaling Technology) overnight at 4 °C. After rinsing, the membrane was incubated for 1 h with alkaline phosphatase conjugated secondary antibody, anti-rabbit IgG, HRP-linked antibody (1:5000, 7074S, Cell Signaling Technology), anti-mouse IgG, or HRP-linked antibody (1:5000, 7076S, Cell Signaling Technology). Finally, the membrane was treated with Super ECL Detection Reagent (YEASEN) and visualized in GE Amersham Imager 600, and the OD of protein bands was quantified using Image J software (https://imagej.en.softonic.com/, Version 1.8.0).

### DAPI staining

Cells were seeded in 6‑well plates at a density of 1 × 10^5^/well (2 ml/well). The cells were treated with quercetin at different concentrations (0, 25, 50 and 100 μM) for 24 h. The medium was removed and 1 mL of 75% alcohol was added to each well, followed by incubation for 10 min. Then, the alcohol was removed, 300 μl of DAPI was added to each well, and representative photos were captured under a fluorescence microscope (ZEISS, Axiocam 506 color).

### Calcein/PI staining

Cells were seeded in 6‑well plates at a density of 2.5 × 10^5^/well (2 ml/well) and then treated with quercetin at different concentrations (0, 25, 50 and 100 μM) for 24 h. Subsequently, cells were stained with Calcein/PI following the manufacturer’s instructions with Calcein/PI Cell Viability Assay Kit. The representative photos were captured under a fluorescence microscope (ZEISS, Axiocam 506 color) and the fluorescence intensity was analyzed.

### Analysis of apoptosis by flow cytometry

Cells were treated with quercetin at different concentrations (0, 25, 50 and 100 μM) for 24 h and subsequently stained with FITC conjugated Annexin V/ propidium iodide (PI). The fluorescence intensity was analyzed with a flow cytometer (BD FACSAria III, USA). The percentage of cells in early apoptosis was calculated as Annexin V-positivity/PI-negativity, while the percentage of cells in late apoptosis was calculated as Annexin V-positivity/PI-positivity. Data analysis was performed with FlowJo software (https://www.flowjo.com/solutions/flowjo, Version vX.0.7).

### Cell cycle analysis

In addition, cells were stained with PI, and flow cytometry was also performed to analyze the cell cycle of EC cells after 1uercetin treatment for 24 h. Data analysis was done with ModFit LT software (https://www.vsh.com/products/mflt/index.asp, Version 3.1).

### ROS detection

The DCFH-DA fluorescent probe was used to determine the intracellular ROS level. Cells were treated with quercetin at different concentrations for 24 h and subsequently stained with DCFH-DA fluorescent probe following the manufacturer’s instructions. The fluorescence was detected by flow cytometer (BD FACSAria III, USA). The representative photos were captured under a fluorescence microscope (ZEISS, Axiocam 506 color).

### Determination of mitochondrial membrane potential

The mitochondrial membrane potential (MMP, ΔΨm) was measured by JC-1 staining. Briefly, cells were treated with quercetin at different concentrations for 24 h and subsequently stained with JC-1 following the manufacturer’s instructions. The fluorescence of JC-1 monomers and aggregates was analyzed, and representative photos were captured under a fluorescence microscope (ZEISS, Axiocam 506 color).

### Statistical analysis

The GraphPad Prism 8.0 (USA) was used for statistical analysis. The t-test was used for the comparation of data between two groups, and one-way analysis of variance (ANOVA) followed by Tukey’s post hoc test was used for the comparation of data among three groups. All the data were obtained from at least three independent experiments and presented as mean ± standard deviation (SD), A value of *p* < 0.05 was considered statistically significant.

## Results

### Quercetin inhibited the proliferation and migration of HEC-1-A cells

As shown in Fig. 1b, quercetin exhibited anti-proliferative effects on the HEC-1-A cells, and the 50% inhibiting concentration (IC50) was 104.2 μM at 24 h and 59.69 μM at 48 h. The Calcein/PI staining was employed to evaluate the effect of quercetin on the activity of HEC-1-A cells. Viable cells showed green fluorescence after calcein AM staining, while dead cells showed red fluorescence. Results showed that the number of dead cells increased significantly with the increase of quercetin concentration (Fig. 1c). As shown in Fig. 1d, the number of colonies formed by HEC-1-A cells treated with quercetin significantly reduced as compared to cells without treatment. This indicates that quercetin inhibits the proliferation of EC cells in vitro. The cell scratch assay also indicated that quercetin markedly inhibited the migration of EC cells (Fig. 1e).

### Quercetin-induced apoptosis of HEC-1-A cells

**Fig. 2:**
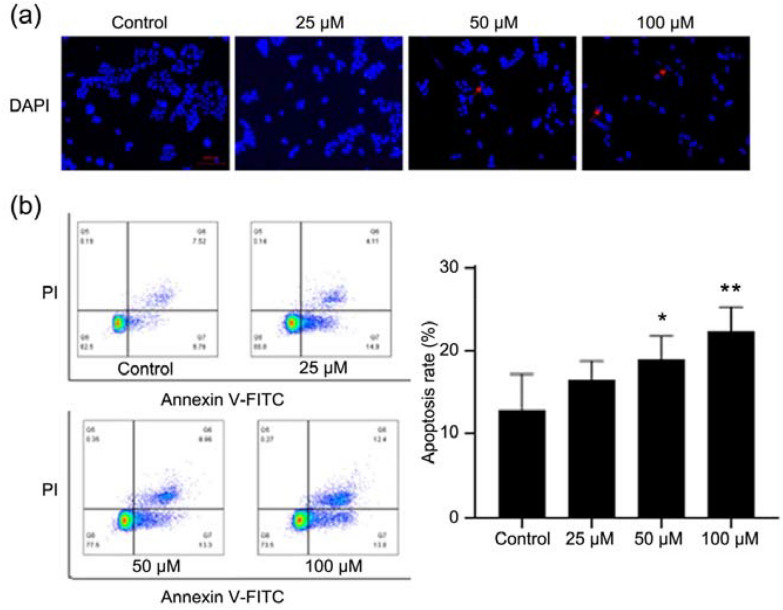
Quercetin induced apoptosis of HEC-1-A cells. **a** Apoptotic assay by DAPI staining. Cells were treated with quercetin for 24 h, and then the apoptotic cells were detected by DAPI staining and visualized under a fluorescent microscope. **b** Cell apoptosis detection by flow cytometry. Cells were treated with quercetin for 24 h, and the apoptotic cells were detected by staining with PI and Annexin V-FITC/PI followed by flow cytometry. Data are expressed as mean ± SD (*n* = 3). *p < 0.05, ** *p* < 0.01 vs. Control

DAPI, a DNA-specific fluorescent dye, is commonly used to detect the nuclear morphology of cells. As shown in Fig. 2a, in the control group, the nucleus of HEC-1-A cells was round and intact with faint fluorescence, while quercetin treatment induced the apoptotic features in the EC cell, such as nuclear pyknosis, deep staining and presence of apoptotic bodies. Furthermore, the cell apoptosis was investigated by FITC-conjugated Annexin V/PI staining via flow cytometry, which is a comprehensively recognized way for cell apoptosis detection. Results indicated that quercetin induced apoptosis of HEC-1-A cells (Fig. 2b).

### Quercetin affected cell cycle of HEC-1-A cells

**Fig. 3:**
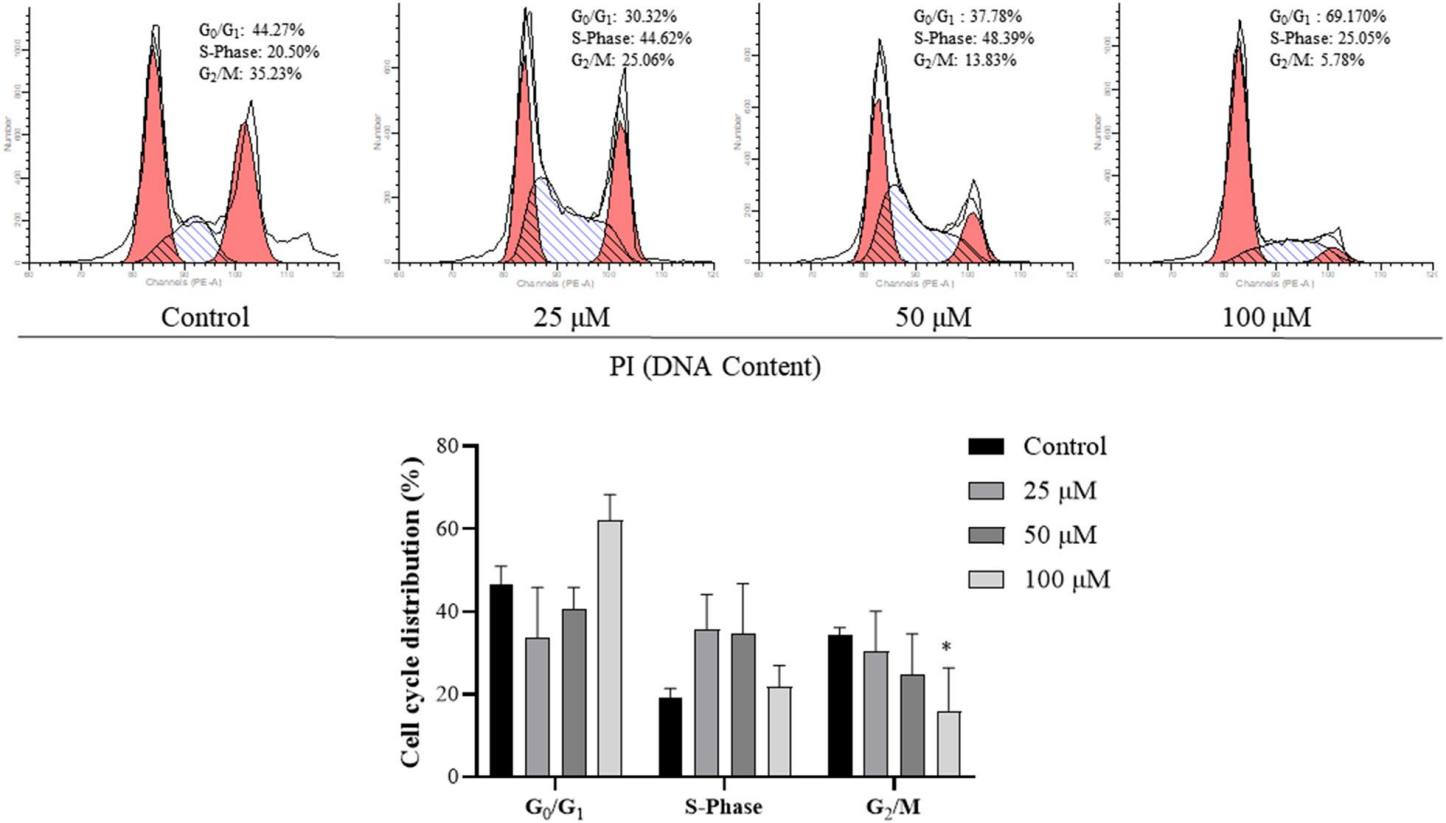
Effect of quercetin on the cell cycle of HEC-1-A cells. Cell cycle detection by flow cytometry. Cells were treated with quercetin for 24 h, then processed and examined with a flow cytometer. Data are expressed as mean ± SD (*n* = 3). *p < 0.05, vs. Control

Cell cycle was further analyzed via flow cytometry. Results showed that the ratio of cells in the G1 phase increased significantly and the ratio of cells in the G2 phase decreased markedly after quercetin treatment (Fig. 3), which means that quercetin prevents cells from entering cell cycle.

### Quercetin induced HEC-1-A cell apoptosis by regulating ferroptosis

**Fig. 4:**
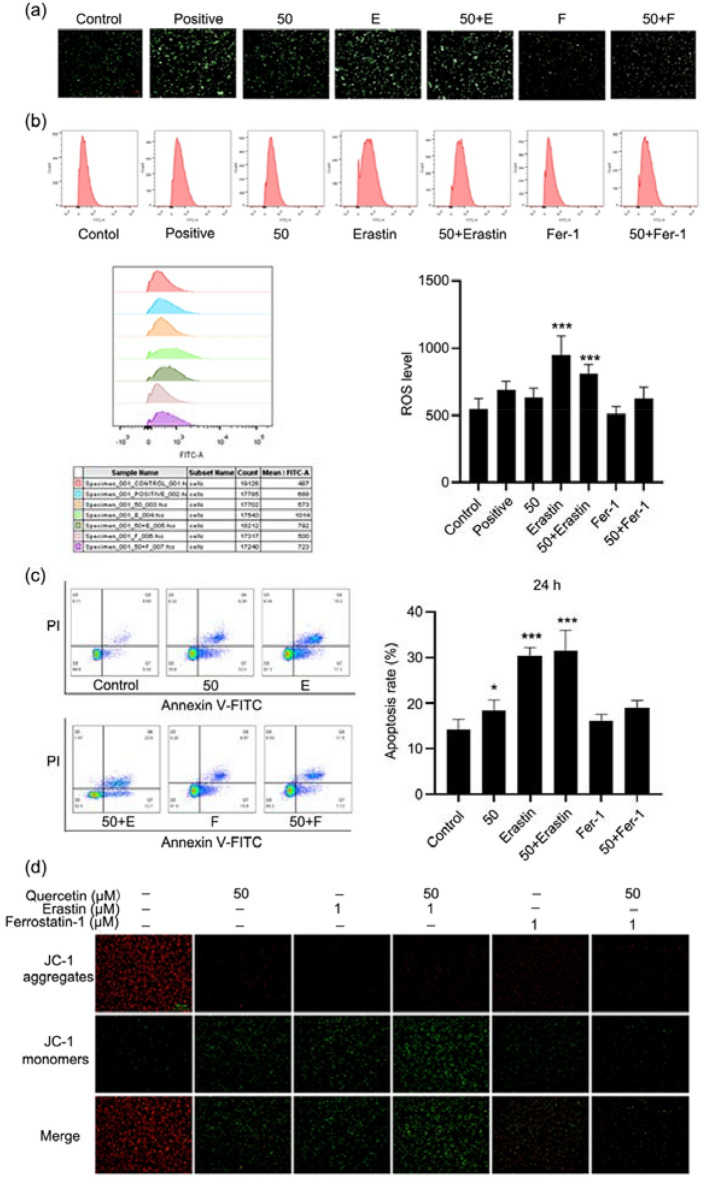
Quercetin induced HEC-1-A cell apoptosis by regulating ferroptosis. **a**, **b** ROS level in HEC-1-A cells. Cells were treated with quercetin for 24 h, then the ROS level was detected by DCFH-DA staining and cells were visualized under a fluorescent microscope and analyzed by flow cytometry. **c** Cell apoptosis detection by flow cytometry. Cells were treated with quercetin for 24 h, and the apoptotic cells were stained with Annexin V-FITC/PI followed by flow cytometry. **d** Detection of mitochondrial membrane potential of HEC-1-A cells by JC-1 staining. Cells were treated with quercetin for 24 h, and visualized under a fluorescent microscope. Red fluorescence represents the mitochondrial aggregate JC-1 and green fluorescence indicates the monomeric JC-1. Data are expressed as mean ± SD (*n* = 3). *p < 0.05, ***p < 0.001 vs. Control

Ferroptosis is a new form of RCD. It is iron-dependent and different from cell apoptosis, cell necrosis and autophagy. The execution of ferroptosis is related to lipid peroxidation and increase in ROS [28–30]. In the present study, HEC-1-A cells were treated with ferroptosis inducer erastin and ferroptosis inhibitor ferrostatin-1 independently, and cells were divided into control group, positive group, quercetin group (50 μM), erastin group (1 μM), quercetin (50 μM) + erastin (1 μM) group, ferrostatin-1 (1 μM) group, and quercetin (50 μM) + ferrostatin-1 (1 μM) group. As shown in Fig. 4a, b quercetin increased the intracellular ROS level in HEC-1-A cells as measured by DCFH-DA staining and flow cytometry. Results showed ferrostatin-1 attenuated the ROS production, and the addition of quercetin further inhibited the ROS production. As shown in Fig. 4c, flow cytometry showed erastin significantly promoted cell apoptosis, and the addition of quercetin further promoted cell apoptosis. However, ferrostatin-1 failed to significantly induce the apoptosis of cells, but the addition of quercetin tended to increase cell apoptosis.

The ΔΨm is also an important indicator of ferroptosis. When the ΔΨm is high, JC-1 is a polymer, and the red fluorescence is observable; when the ΔΨm is low, JC-1 is a monomer, and the green fluorescence may be observable. As shown in Fig. 4d, compared with control group, quercetin significantly reduced the ΔΨm, and the combination of quercetin and erastin could exert synergistic effect to reduce the ΔΨm. These results indicate that quercetin induces apoptosis of EC cell by regulating ferroptosis.

### Quercetin affected ferroptosis related pathways in HEC-1-A cells

**Fig. 5:**
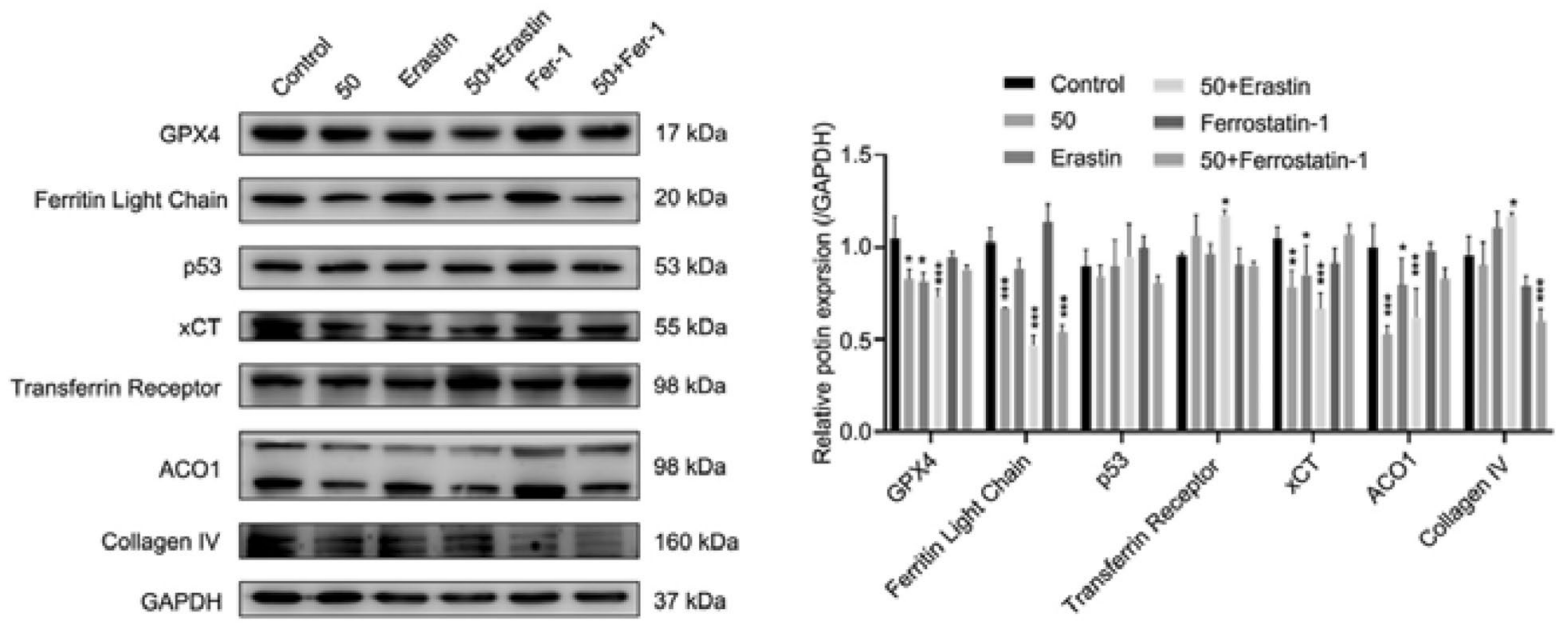
Effect of quercetin on ferroptosis related pathways in the HEC-1-A cells. Effects of quercetin on the ferroptosis related proteins. Data are expressed as mean ± SD (*n* = 3). *p < 0.05, **p < 0.01, ***p < 0.001 vs. Control

The expression of molecules in the ferroptosis-related pathways was detected through Western blotting (Fig. 5). Glutathione peroxidase 4 (GPX4) is the most important anti-lipid peroxidase in cells and a core regulator of ferroptosis. Our results showed the GPX4 expression in the quercetin group and erastin group was significantly lower than in the control group (*p* < 0.05), and the combination of quercetin and erastin further decreased GPX4 expression (*p* < 0.001). xCT, also known as Slc7a11, is an important antioxidative protein in the mammals and involved in the cystine transport pathways. Our results revealed that quercetin or erastin treatment could significantly upregulate the xCT expression (*p* < 0.05), and the combination of quercetin and erastin synergistically increased the xCT expression. In addition, the expression of both ferritin light chain and anti-aconitase 1 reduced markedly in the quercetin and erastin group (*p* < 0.001), the expression of transferrin receptor and collagen IV increased significantly after treatment with both quercetin and erastin as compared to control group (*p* < 0.05). p53 is also an important tumor suppressor gene. In our study, quercetin failed to significantly change the expression of p53.

## Discussion

In recent years, a large number of studies have confirmed that quercetin at a reasonable concentration has a variety of biological activities, such as anti-inflammatory, anti-oxidant, and anti-cancer activities. It has been added to functional foods as a commercial dietary supplement, and increasing studies have revealed that quercetin may be employed to prevent or treat various diseases such as cancers [31, 32]. To date, few studies have conducted to investigate the effect of quercetin on the EC. The present study was undertaken to preliminarily investigate the biological effects of quercetin on the EC cells (HEC-1-A cells), and the role of ferroptosis in these effects was further explored in vitro. CCK-8 assay showed quercetin significantly inhibited the proliferation of HEC-1-A cells, and the IC_50_ was 104.2 μM at 24 h and 59.69 μM at 48 h. PI/ Calcein AM fluorescence staining further confirmed the inhibitory effect of quercetin on the HEC-1-A cells. The colony formation assay indicated quercetin inhibited the colony formation of HEC-1-A cells, and the cell scratch assay showed quercetin suppressed the migration of HEC-1-A cells. All these findings confirm that quercetin is able to significantly inhibit the proliferation and migration of HEC-1-A cells. Then, the effect of quercetin on the apoptosis of HEC-1-A cells was further studied. Both flow cytometry and DAPI staining indicated that quercetin at different concentrations induced the apoptosis of HEC-1-A cells. Moreover, after quercetin treatment, flow cytometry showed the ratio of cells in the G1 phase increased markedly but the ratio of cells in the G2 phase decreased significantly, which means that quercetin prevents cells from entering the cell cycle.

In the following experiments, the mechanisms by which quercetin induces EC cell death were explored. Ferroptosis is a new form of RCD and is mainly manifested by iron dependent lipid peroxidation. The ferroptosis inducer (erastin, Era) and/or ferroptosis inhibitor (ferrostatin-1, Fer-1) was used to treat EC cells with or without quercetin treatment. Interestingly, our results showed quercetin or erastin treatment increased ROS production as shown by flow cytometry and *in-situ* fluorescence staining, and the combination of quercetin and erastin could exert synergistic effect on the ROS production. Fer-1 treatment had little influence on the ROS production. In addition, the number of apoptotic cells increased significantly in the quercetin group and Era group, and quercetin and erastin synergistically increased the apoptotic cells, while the number of apoptotic cells remained unchanged in the Fer-1 group as compared to the control group. The changes of ΔΨm was further detected after quercetin treatment. The results showed that quercetin or erastin treatment decreased the ΔΨm significantly, and JC-I changed from a polymer state to a monomer state after quercetin or erastin treatment, while the ΔΨm in the Fer-1 group was at a high level, and JC-1 was mostly in a polymer state. The expression of some key molecules in the ferroptosis pathway was also detected by Western blotting. GPX4 is the most important anti-lipid peroxidase in cells, and also the core regulator of ferroptosis [33]. xCT, also known as Slc7a11, is an important antioxidative protein in the mammals. xCT mainly acts to promote the production of intracellular glutathione, eliminate the damage of ROS to the cells, and protect the cell survival. ACO1 (also known as IREB1) is an iron regulatory protein, and acts as an iron sensor. Our results indicated that both quercetin and erastin treatment significantly reduced the expression of GPX4, xCT, ACO1 and ferritin light chain, and the expression of transferrin receptor markedly increased. In recent years, it had been reported that p53 mediated ferroptosis plays an important role in the tumor inhibition. Jiang et al. [34] found that p53 inhibited the transcription of SLC7A11 gene through 5’ side region p53 response element. After reducing the expression of SLC7A11 and inhibiting the uptake of cystine through the p53 pathway, the sensitivity of cell ferroptosis increased. Although our results showed that quercetin had no significant effect on the expression of p53 in the HEC-1-A cell, ferroptosis may be regulated by other pathways. Iron is taken up as ferrous and deposited as iron hydroxide after oxidation. The ferritin light chain (FLC) plays a role in the delivery of iron to cells and mediates iron uptake in the developing renal tunicate cells [35, 36]. Cellular uptake of iron occurs via receptor-mediated endocytosis of ligand-occupied transferrin receptor into specialized endosomes. Endosomal acidification leads to iron release. Transferrin receptors are required for the development of red blood cells and the nervous system [37, 38]. The results showed that quercetin inhibited the expression of FLC and had no significant effect on the expression of transferrin receptor, but quercetin combined with erastin increased the expression of FLC. We also examined the expression of Collagen IV, which is known to inhibit angiogenesis and tumor formation, and the results showed that quercetin had no significant effect on it.

In recent years, it has been found that the ferroptosis is induced in cancer cells, including colon cancer [39], gastric cancer [40], lung cancer [41] and triple negative breast cancer [42], and the combination of ferroptosis activators and anti-tumor drugs is beneficial for the tumor intervention and inhibition. Guo et al. [43] found that Honokiol (HNK) reduced the viability of colon cancer cell lines by increasing ROS and Fe^2+^ levels, and induced the ferroptosis of these cells by reducing GPX4 activity. As a potential therapeutic drug, HNK shows good anti-cancer effects through multiple signaling pathways. A study for the first time reveals that metformin is able to induce ferroptosis, which may be a new mechanism underlying the anti-cancer effect of metformin [44]. SLC7A11 is a new ubiquitin-fold modifier (UFM) methylation substrate, and metformin reduces the protein stability of SLC7A11 by inhibiting the UFM-yylation process of SLC7A11. SLC7A11 is an important ferroptosis regulator. Metformin combined with sulfapyridine as a systemic Xc-inhibitor can synergistically induce ferroptosis and inhibit the proliferation of breast cancer cells. The process of ferroptosis involves the expression of many genes and regulation of different signaling pathways. Therefore, it is of great significance to further study the molecular mechanism of ferroptosis and explore its role in different diseases, which may provide evidence for the development of anticancer drugs.

A large number of studies have proven that ferroptosis induction can inhibit cancer growth and exert anti-cancer effect [23, 45]. On one hand, drugs that can promote ferroptosis may be employed to eliminate cancer cells or virus-infected cells. On the other hand, healthy cells should be protected by inhibiting ferroptosis. The Traditional Chinese Medicine has abundant resources for the development of anti-cancer drugs because there are a variety of active ingredients in the Traditional Chinese Medicine that may exert a regulatory effect on the ferroptosis [46, 47]. The use of small molecule compounds to regulate ferroptosis has become a hot topic in medical studies on the treatment of diseases. Based on network pharmacology, Ou et al. screened out a number of ferroptosis-related targets, such as quercetin, apigenin, luteolin and other known small molecular compounds of traditional Chinese medicine. Among the 15 potential targets of iron death, seven targets showed strong sensitivity to traditional Chinese medicine [48]. It also provided a thought for the future clinical integration of traditional Chinese and western medicine and the development of ferroptosis intervention drugs.

Our study, for the first time, investigates the role of ferroptosis in the anti-cancer effects of quercetin on EC cells. However, there were limitations in our study. Only one cell line of EC was investigated in this study; the anti-cancer effect of quercetin was not confirmed in animal models; the specific ferroptosis related signaling pathway regulated by quercetin was not further investigated. Taken together, our study indicates that quercetin inhibits cell growth by inducing ferroptosis in the HEC-1-A cells, which may provide a new and novel therapeutic strategy for the treatment of EC. Although our study is only preliminary, it provides a new direction for the treatment of EC and presents a basis for the development of therapeutic drugs for EC. However, further studies will be carried out, including in vivo experiments, and the specific mechanism by which ferroptosis affects the malignant behaviors of EC should be investigated extensively.

## Conclusions

In conclusion, our study shows quercetin can inhibit the proliferation and migration of EC cells, which may be related to the regulation of ferroptosis. This study explores the mechanism underlying the inhibitory effect of quercetin on the growth of EC cells and provides a reference for the development of drugs for the treatment of EC in the future.

## Data Availability

The datasets used and analyzed during the current study are available from the corresponding author on reasonable request.

## Abbreviations

EC: Endometrial carcinoma
RCD: Regulated cell death
ROS: Reactive oxygen species
NR-κB: Nuclear factor-kappa B
MAPK: Mitogen-activated protein kinase
AMPK: AMP-activated protein kinase
CCK-8: Cell-Counting-Kit-8
ACO1: Anti-aconitase 1
HRP: Horse radish peroxidase
OD: Optical density
PI: Propidium iodide
GPX4: Glutathione peroxidase 4
HNK: Honokiol
UFM: Ubiquitin-fold modifier
